# Long-term care insurance, mental health of the elderly and its spillovers

**DOI:** 10.3389/fpubh.2023.982656

**Published:** 2023-03-03

**Authors:** Yunfei Chen, Hong Zhao

**Affiliations:** ^1^School of Economics, Shanghai University, Shanghai, China; ^2^School of Economics, Qingdao University, Qingdao, China

**Keywords:** long-term care insurance, aging population, mental health, spillover effects, China

## Abstract

The paper studies the effects of the long-term care insurance (LTCI) program in China on the mental health of older adults and the wellbeing of their families. We employ the staggered difference-in-differences approach based on the LTCI pilots from 2015 to 2017. First, we find the LTCI program improves older adults' happiness and reduces depression symptoms significantly. The effects on the improvement in memory and cognition are associated with the elderly with activities of daily living-related need for care. Second, the effects of LTCI are partially mediated through providing community services, relieving care burdens, and reducing the incidence of diseases. More importantly, LTCI coverage improves caregivers' physical health and social activities, reflecting its welfare spillover effects. Furthermore, the relationship between LTCI and mental health differs due to the difference in LTCI designs and older adults' demographic characteristics. This presents a need to consider mental health in the services and evaluation criteria of LTCI.

## 1. Introduction

Rapid aging has become a worldwide challenge, which has led to marked demands for medical and nursing care, and great financial pressure, especially in the post-COVID-19 era (1). The unmet long-term care has received much attention (2, 3). China, with the largest elderly population over 65, faces greater challenges. China's population aged 65 and over has reached 191 million, accounting for 13.5% of total population.^1^ It is expected to reach 366 million by 2050, rapidly advancing toward an aging society.^2^ In addition, more than 78% of older people are suffering from chronic disease, mental illness, cognitive and sensory decline or other problems. People living with dementia in China account for nearly 20% of the world (4). The number of older people with needs for activities of daily living–related (ADLs) care is growing rapidly (1). However, traditional care in China provided by older adults' family members is not enough due to smaller families and delayed fertility (5, 6). Also, it is generally of relatively poorer quality (7), which is detrimental to the rehabilitation of older people and has adverse effects on the wellbeing of their caregivers (8, 9).

In response to these challenges, some OECD countries, such as the Netherlands in 1968, the United States in the 1970s, Germany in 1995, and Japan in 2000, have launched the long-term care insurance (LTCI) program (10, 11). The program enables older people to obtain care services and health care, however, universal problems do exist such as insufficient services, unfairness and narrow coverage (12, 13). Considering surging demands for care and inadequate resources, China started the LTCI program in Qingdao in 2012, and officially launched staggered LTCI pilots in 15 cities during 2016 and 2018. By 2021, China's LTCI program has covered more than 140 million people in 49 pilot cities. China's aged care market is still underdeveloped. Although older adults covered by LTCI are more easily accessible to health care, the diagnosis and management of mental health are inadequate (14). Therefore, studying the impact of the pilot practices of China's LTCI on older adults' mental health is not trivial, not only because LTCI plays an important role in coping with the mounting and various demands for care, but also empirical evidence on how China responds to rapid aging can provide suggestions for middle-income countries with rapidly aging population to promote, design and reform their LTCI program.

Our paper has several contributions as follows. First, there is little systematic, theoretical or empirical study on the effect of LTCI on comprehensive mental health. This study adds to the emerging evaluation of LTCI programs worldwide. From subjective and objective perspectives, we examine the effects and influencing mechanism of LTCI on the mental health of insured people and those with the need for ADLs care. The mental health of older adults should be paid more attention, because they are more likely to have reduced cognitive abilities and psychological decline with aging, and these changes may further lead to depression. Mental health disorder has become a major problem for older adults (6). Despite the importance of this issue, it remains unclear whether LTCI could improve mental health. Extant studies, merely covering one aspect of mental health, may not be enough to assess the mental health status of older adults. Mental health not only indicates the absence of psychiatric disorders, but also a good cognitive and response function and welfare state according to the definition of the WHO (15). In this regard, we construct four mental indexes, including objective health (memory and cognitive function) and subjective health (depressive symptoms and life satisfaction) to provide a more comprehensive assessment of the effects of LTCI on the elderly's mental health.

Second, we study the staggered LTCI policy implemented in 15 cities from 2015 to 2017 based on the difference-in-differences (DID) approach, and we explore whether and how China's LTCI affects older adults' mental health. On the one hand, China, as the largest developing country, has the largest elderly population in the world. A better understanding of China's LTCI can offer experiences to other developing countries with the same challenge of aging populations and care burdens. On the other hand, the study on China's LTCI is still limited due to its shorter implement time. The recent literature on China's LTCI only studies one pilot city such as Qingdao and Shanghai, or LTCI programs launched in the same year (16–18). Overall, there is still no consensus regarding the impacts of LTCI on mental health, especially in China, and merely considering one pilot or a specific type of health outcome might not capture the relationship between LTCI and mental health. This paper appears to be the first study to investigate China's staggered LTCI policy in the initial 15 pilot cities from 2015 to 2017, and it can provide a more comprehensive overview of the effects of the program. The assessment of the effects and the study on the channels through which China's LTCI influence mental health also have policy implications for promoting the reform and expanding LTCI pilots.

Third, the welfare spillover effects of LTCI on their families especially their caregivers need to be further studied. In fact, some literature documented that caregivers tend to report a lower quality of life and a deterioration of mental health compared to non-caregivers (5, 19, 20). Therefore, this study examines not only the mental health of the covered older adults but also the wellbeing of their spousal caregivers who is the main caregiver in China. Spousal caregivers suffer from higher level of stress than children caregivers (21), and have a common problem of social isolation (22). Accordingly, we examine the effects of LTCI on their self-reported health, life satisfaction and social activities. The findings in our paper offer useful insights to expand beneficiaries.

Finally, we try to figure out the heterogeneity of the relationship between LTCI and mental health. The results of our study confirm LTCI designs (the eligibility for LTCI and financing standards), socioeconomic and demographic differentials (gender, urban or rural, family income, marital status, education, and the family size) in the effects of LTCI on the mental health of older people. Our findings will help policymakers craft appropriate strategies to design and reform China's LTCI system, and eventually improve older adults' mental health and wellbeing.

The rest of the paper is organized as follows. Section 2 presents the background of China's LTCI, and Section 3 is literature review. We show the theoretical influence mechanism, empirical methodology and data in Section 4. Section 6 presents and discusses the empirical results. Section 6 concludes and provides policy implications.

## 2. Literature review

A large amount of literature has studied the LTCI program in developed countries, including the effects on care recipients' health (23), hospital stay and expenditure (24, 25), the choice of care (26), financial burdens (27), their families (28) and national economic welfare (29). Among these, however, the findings on health outcomes are contradictory. For instance, Lei et al. (4) and Sohn et al. (30) found that LTCI is correlated with better self-reported health and lower mortality risk, while Kim and Lim (27) and Fu et al. (31) argued that LTCI has a limited effect on older adults' health. These inconsistent findings can be partly attributed to the difference in countries' socio-economic characteristics, the LTCI program's key features and its implementation time. In particular, the assessment of the social and economic impacts of developing counties is limited. The implementation of LTCI in China has attracted growing interest from many scholars. For example, Lu et al. (17) and Feng et al. (16) found that China's LTCI significantly reduces medical expenditures and health insurance expenditures. In addition to the reduction in financial burdens, Lei et al. (18) further found that LTCI improves older people's self-reported health and reduces their mortality risks. However, these studies mainly focus on only one pilot city (13, 32). The study on China's LTCI is still not enough, especially the limitation on older adults' mental health and spillover effects. Our study will address this gap.

In addition to its far from conclusive, extant literature on mental health outcomes is not comprehensive not only in the definition of mental health but also in affected members. On the one hand, previous studies only focused on one aspect of mental health, such as depression symptoms (18), which may not be enough to assess the mental health status of older adults. For instance, Tang et al. (33) found the positive effects of LTCI on mental health measured by older adults' depression symptom. According to the definition of the WHO (15), however, mental health includes the absence of psychiatric disorders and a good cognitive and response function. Yet to date, there is no systematic and empirical evidence on the influence of LTCI on mental health. On the other hand, many researches have sought to give answers to the spillover effects of LTCI. Fu et al. (31) found that LTCI is associated with increased labor supply. Costa-Font and Vilaplana-Prieto (34) documented that caregiving supports could alleviate depressive symptoms of caregivers. However, extant literature has not yet reached a consensus on the effects on caregivers, especially their spouse who are main caregivers and are more likely to suffer from social isolation and depressive symptoms (20, 21). Our focus on caregivers' wellbeing, including life satisfaction, physical health and social participation, complements previous related literature focusing on LTCI's spillover effects (35).

## 3. Background

Rapid aging, causing lower employment rate and increased demands for medical and nursing care, has become a social problem in China. China has established medical insurance for urban workers (the Urban Employees Basic Medical Insurance, UEBMI), urban residents (Urban Resident Basic Medical Insurance, URBMI), rural population and other people (Urban and Rural Residents Basic Medical Insurance, URRBMI). Although these medical policies share parts of medical costs, older adults' needs for medical or nursing care are unmet. In response to the challenge, Chinese government has decided to establish its independent social insurance to provide socialization of care, relatively separating from previous medical insurance. China officially introduced the LTCI pilots in 15 cities in 2016 and further added 34 pilots in 2020 (36). Qingdao as one of the 15 LTC programs has started the long-term care insurance pilots since 2012 and conducted some reforms in 2015 (37). The LTCI system has covered more than 140 million people in 49 pilot cities by 2021.

We study the initial 15 pilot cities, key features of which are shown in Table 1. China's LTCI system is mainly financed by public medical insurance pooled funds, together with fiscal subsidies, employer contributions and welfare lottery funds (38). It mainly covers people enrolled in UEBMI. Some pilots, such as Qingdao, Changchun and Jingmen, also cover residents enrolled in URRBMI and URBMI. The access to care service is primarily determined by the covered people's disability degree, mostly evaluated by the Barthel scale for ADLs. Some pilot cities also include other factors such as cancer and dementia. As shown in Table 2, care recipients in China are provided with three categories of care services that are home-visit care, community, and institutional care services (designated medical institutions, nursing institutions and aged care institutions). The reimbursement rate mainly depends on the type of services, and the majority covers ~70–90% of the costs, with a payment ceiling up to 20–60 yuan per person. It is worth noting that the LTCI of Shanghai not only provides care services but also cash benefits.

**Table 1:** Key features of 15 pilot cities.

**City**	**Date**	**The covered area**	**Insured population**	**Eligibility**
Anqing	2017-1	Urban areas	UEBMI	Severe disability
Changchun	2015-3	All	UEBMI; URBMI	Severe disability/advanced cancer
Chengde	2017-1	Urban areas	UEBMI	Severe disability
	2018-12	All		
Chengdu	2017-7	All	UEMBI	Severe disability
Chongqing	2018-1	Dadukou, Banan, Shizhu, Dianjiang	UEBMI	Severe disability
Guangzhou	2017-8	All	UEBMI	Severe disability; dementia plus moderate disability
Jingmen	2016-11	All	UEBMI	Severe disability
	2017-1	All	URRBMI	
Nantong	2016	Urban areas	UEBMI; URBMI	Severe disability; moderate disability
	2019	Haimen, Qidong	URBMI	
Ningbo	2017-12	Urban areas	UEBMI	Severe disability
Qingdao	2012-7	All	UEBMI; URBMI	Disability level 3–5; dementia
	2015-	All	URRBMI	
Qiqihaer	2017-10	Urban areas	UEBMI	Severe disability
Shanghai	2017-7	Xuhui, Putuo, Jinshan	UEBMI; URRBMI	Aged >60 years; disability level 2–6
Shangrao	2017-7	All	UEBMI	Severe disability
	2019	All	UEBMI; URRBMI	
Shihezi	2017	Urban areas	UEBMI; URRBMI	Severe disability
Suzhou	2017-6	All	UEBMI; URRBMI	Severe and moderate disability

**Table 2:** Care services and benefits package of 15 pilot cities.

**City**	**Home-visit/community**	**Aged care/nursing institutions**	**Medical institutions**
Anqing	Cap 750/m	Co-payment 50%; cap 40/d	Co-payment 60%; cap 50/d
Changchun	–	Co-payment 90% (UEBMI); 80% (URBMI, URRBMI)	
Chengde	70%; cap 40/d	Co-payment 70%; cap 50/d	Co-payment 70%; cap 6/d
Chengdu	Co-payment 75%	Co-payment 70%	Co-payment 70%
Chongqing	Cap 50/d		
Guangzhou	90%; cap 115/d	75%; cap 20/d	75%; cap 1,000/m
Jingmen	80%; cap 100/d (full time); cap 40/d (part time)	75%; 100/d	70%; 150/d
Nantong	Cap 1,200/m	Co-payment 50%	Co-Payment 60%
Ningbo	–	Cap 40/d	Cap 40/d
Qingdao	In 2012: home-care 60/d, 96%; professional care 170 /200/d, 90%; In 2015: home/community/professional care 50/65/170/d; 90% (UEBMI); 80% (children/level 1), 40% (level 2)		
Qiqihaer	50%; cap 20/d	55%; cap 25/d	60%; cap 30/d
Shanghai	Home-visit care 90%; community services 85%;	Co-payment 85%	Co-payment 90%
Shangrao	Family care 450/m; home-visit care 900/m	Cap 1,200/m	–
Shihezi	Cap 25/d; cap 750/m	70%; cap 750/m	70%; cap 750/m
Suzhou	Cap 30/d (severe disability); CAP 25/d (moderate disability)	cap 26/d (severe disability); cap 20/d (moderate disability)	

“Cap” means the daily benefit cap; “/d” means yuan per person per day; “/m” means yuan per person per month.

## 4. Theoretical and empirical analysis

### 4.1. Theoretical framework

**Figure 1:**
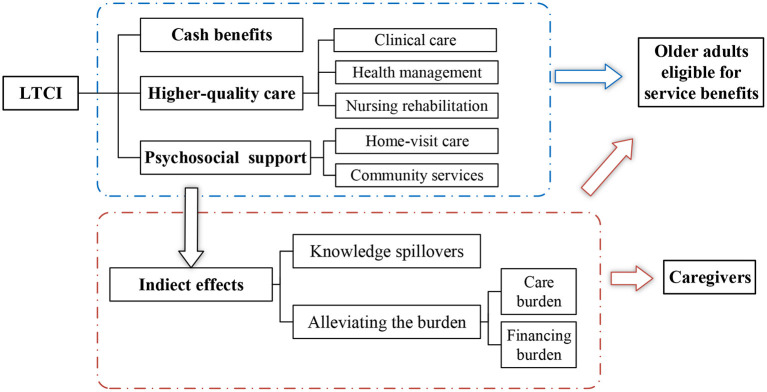
Theoretical framework.

As shown in Figure 1, the LTCI system influences subjective and objective mental health in four ways. First, the LTCI program in China provide higher-quality care, effective health management and nursing intervention, which could improve older adults' memory and cognitive function (39, 40). To meet the needs for rehabilitation, LTCI also offers better follow-up which is useful to maintain older people's physical and mental health. Second, home-visit care and community services provided by LTCI improve older people's psychosocial contact and support. These services can alleviate their loneliness and depression (18), eventually improving their life satisfaction (41). Third, the promotion of LTCI in China provides much knowledge about health management for an aging population. It not only benefits their physical health through self-rehabilitation but also helps them regulate depression symptoms. Fourth, this long-term care service is a substitute for informal care by family members and expensive institutional care (27, 42). LTCI covers most of care services costs, even in some pilot cities where LTCI provides cash benefits. Therefore, LTCI may improve older adults' mental health by alleviating the psychological burden of expected financial and care costs.

Family members of disabled older people as caregivers are more likely to feel a loss of independence and freedom (43, 44), thus their mental health deteriorates. LTCI plays an important role in the wellbeing of older people's caregivers (45). On the one hand, LTCI has proved a substitute for informal care which releases caregivers' care responsibilities and disposable time (34). The reduction in care burdens gives caregivers opportunities to pursue other activities (46), and increases the sense of personal control over their life (47), thus lowering the prevalence of depression and improving their life satisfaction (48, 49). On the other hand, LTCI is hypothesized to benefit caregivers' physical health by providing health care knowledge and improving their self-care and proactive health management. Based on these considerations, LTCI can also offer significant benefits to caregivers' physical health.

### 4.2. Model and methods

We construct the staggered difference-in-differences (DID) model which can reduce the endogenous biases and estimate more accurately to study the effects of LTCI on mental health:


(1) $$\begin{array}{l} Y_{ijt}=\beta_{0}+\beta_{1}LTC_{ijt}+\beta_{2}X_{ijt}+\beta_{3}Z_{jt}+{\text{λ}}_{i}+\gamma_{t}+\varepsilon_{ijt} \end{array}$$

where *Y*_*ijt*_ represents mental health outcomes of individual *i* living in city *j* in year *t*, including their memory and cognitive function, depression symptoms and happiness. We choose six pilot cities from the initial 15 pilot cities officially announced in 2016, because they covered persons in the whole city before 2018, which are Qingdao, Chengdu, Guangzhou, Jingmen, Shangrao, and Suzhou. Older adults in other 98 cities that never implement LTCI program and non-covered people in pilot cities during the sample period are defined as control groups. *LTC*_*ijt*_ is measured by *treat*_*ijt*_ × *post*_*jt*_. *treat*_*ijt*_ is a dichotomous variable indicating the treated status. *treat*_*ijt*_ = 1 means people enrolled in the medical insurance required by pilot cities. *post*_*jt*_ is a dummy variable (*post*_*jt*_ = 0 for 2011, 2013 and for 2015, 2018 in Qingdao, *post*_*jt*_ = 0 for 2011, 2013, 2015, and *post*_*jt*_ = 1 for 2018 in other cities). *X*_*ijt*_ is a vector of older adults' time-varying characteristics including their age, marital status, children and physical health status. *Z*_*jt*_ is a vector of regional characteristics measured by GDP per capita and fiscal expenditure.λ_*i*_ is the individual fixed effects that absorb time-invariant factors such as the respondent's gender, education, treated status (*treat*_*ijt*_), and other unobservable variables. γ_*t*_ represents year fixed effects.

The access to care service largely depends on covered people's degree of disability, but the CHARLS data has no direct information on current care recipients. Therefore, our estimates are “intention to treat” effects (ITT), indicates overall effects on the targeted population (50). Since ITT is inclined to underestimate the average treatment effect (ATT), our findings are more convincing (51). Moreover, to strength our results, we use the difficulties in ADLs to represent whether the covered older people receive care services. We provide indirect evidence of the different effects of LTCI on the insured people with needs for ADLs care compared to those without needs. We add the interaction term *LTC*_*ijt*_ × *ADL*_*ijt*_. *ADL*_*ijt*_ = 1 indicates the elderly have difficulties in at least one activity among bathing, eating, dressing, moving, and using toilets:


(2) $$\begin{array}{l} Y_{ijt}=\alpha_{0}+\alpha_{1}LTC_{ijt}+\alpha_{2}LTC_{ijt}\times ADL_{ijt}+\alpha_{3}ADL_{ijt}+\alpha_{4}ADL_{ijt}\\ \;\times \;treat_{ijt}+\alpha_{5}ADL_{ijt}\times post_{ijt}+\alpha_{6}X_{ijt}+\alpha_{7}Z_{jt}\\ \;+{\text{λ}}_{i}+\gamma_{t}+\nu_{ijt} \end{array}$$

According to influencing mechanisms of LTCI on mental health, we further choose four mediating variables to identify the effect path, including community services (health check-ups and entertainment in the community), care burdens of older people's children and the incidence of diseases. Following previous studies (52), we adopt the mediating effects model:


(3) $$\begin{array}{lll} M_{ijt} & = & \rho +\rho_{1}LTC_{ijt}+\rho_{2}X_{ijt}+\rho_{3}Z_{jt}+\lambda_{i}+\gamma_{t}+\varsigma_{ijt} \end{array}$$

(4) $$\begin{array}{l} Y_{ijt}=\omega_{0}+\omega_{1}LTC_{ijt}+\omega_{2}M_{ijt}+\omega_{3}X_{ijt}+\omega_{4}Z_{jt}+\lambda_{i}+\gamma_{t}\\ \;+\xi_{ijt} \end{array}$$

Where *M*_*ijt*_ is the mediating variable. If both ρ_2_ and ω_2_ are statistically significant, *M*_*ijt*_ can be regarded as a mediator of the effects of LTCI on mental health.

### 4.3. Data

We use the China Health and Retirement Longitudinal Study (CHARLS) in 2011, 2013, 2015, and 2018 (http://charls.pku.edu.cn/). The data randomly selects 150 counties from 28 provinces in China and collects comprehensive individual and family characteristics of older people aged 45 and above, as representative of China's mid-age and elderly. Considering that age is a primary indicator of care needs, the higher the age, the greater the possibility of disability (16). We choose older adults over 65 years, who are more likely to receive care services, and 4,962 samples are finally used. Table 3 represents the definition and descriptive statistics of variables. Mental health indexes in our paper consist of older people's objective and subjective mental health. Objective mental health index is biomarkers calculated by questions measuring older people's the memory and cognitive function (*Memory* and *Cognition*). Subjective mental health is self-reported measures about biomarkers older adults' subjective feeling (*CES*−*D* and *Happiness*). The mean memory index is 7.310, showing the impaired memory function of older adults in China. The elderly's cognition is better according to the mean is over 8. The average age of older people is 69.166 years, 62.3% of whom are men. The education level of them is low with average 6.7 years of schooling. Most older adults in our study sample have a spouse (83.6%) and have three children on average. Table 4 shows older adults' choice of types of care. We observe that their spouse and children (grandchildren) are the main caregivers.

**Table 3:** Descriptive statistics.

**Variable**	**Definition**	**Mean**	**Std. dev**.
**Dependent variables**			
Memory	20 questions about immediate and delayed recall [0, 20]	7.310	3.360
Cognition	10 questions about calculation and orientation [0, 10]	8.002	1.930
CES-D	Older adults' feeling and behaviors [0, 30]	7.292	5.786
Happiness	Life satisfaction: the higher the value, the better it is [0, 5]	3.267	0.691
Shealth	Caregivers' self-reported health: the higher the value, the healthier it is [0, 5]	2.888	0.963
Shappiness	Caregivers' life satisfaction: the higher the value, the better it is [0, 5]	3.261	0.768
Social	Do social activities (interacted with friends; Play Ma-jong/chess/cards; go to community club) = 1; others = 0	0.382	0.486
**Mediating factors**			
Care	Receive care by their children = 1	0.084	0.277
Disease	The number of diseases including cancer, stomach/digestive disease, asthma, and dyslipidemia	0.589	0.721
Checkup^a^	Receive regular medical examination=1; others = 0	0.210	0.408
Entertain	Entertainment from community care services=1; others = 0	0.024	0.153
**Control variables**			
Age	Older adults' age (years)	69.166	5.074
Gender	Male = 1; female = 0	0.623	0.485
Edu	Years of schooling (years)	6.706	3.950
Agri	Agricultural hukou = 1; others = 0	0.642	0.480
Married	Married =1; others = 0	0.836	0.370
Children	The number of older adults' children alive	3.185	1.387
GDP	GDP per capita of the city (million RMB)	449.459	603.280

GDP per capita and fiscal expenditure are adjusted to eliminate the price effect with 2010 as the base period.

a The information of community serves (check-up and social) is only available in the 2018 CHARLS.

**Table 4:** Types of care.

**Types**	**Spouse**	**Children (grandchildren)**	**Other relatives**	**Institution**
Numbers	2,276	2,035	83	53
All^a^	3,991	10,466	3,952	5,758
Proportion	57.03%	19.44%	2.10%	0.92%

a The number of older people who answered the question in CHARLS data.

## 5. Results and discussions

### 5.1. Main effects on mental health

Table 5 presents the effects of LTCI on the subject and the objective mental health of the elderly. LTCI coverage is recognized as an important role in reducing the insured people's depression and improving their happiness. It is associated with reductions in the score of CES-D by 1.616, and increases in their life satisfaction by 0.109. Attributed to expected reduction in care burden, financial strain and loneliness, the subjective mental health of older people increases. However, a negative impact is found on older adults' memory function and no impact is observed on their cognition function. The reason may be that there exists a moral hazard. Although LTCI could ease older people's fears of care burdens in the future, it also allows them to heavily depend on LTCI, lack the sense of responsibility and reduce health management (13). In addition, Table 5 shows the effects of control variables on older adults' mental health. Older adults who have married experience less memory and cognitive decline and have higher levels of life satisfaction. Better physical health of older people could increase their life satisfaction.

**Table 5:** The effects of LTCI on older people's mental health.

**Variables**	**Memory**	**Cognition**	**CES-D**	**Happiness**
LTC	−0.820^*^ (0.460)	−0.151 (0.200)	−1.656^**^ (0.617)	0.112^***^ (0.023)
Age	−0.209 (0.316)	0.231^*^ (0.116)	0.107 (0.377)	0.009 (0.042)
Agri	0.005 (0.353)	0.084 (0.136)	0.689 (0.499)	0.046 (0.057)
Married	0.827^***^ (0.258)	0.262^*^ (0.146)	−0.163 (0.500)	0.107^**^ (0.046)
Children	−0.144 (0.128)	−0.112 (0.075)	0.082 (0.183)	−0.004 (0.018)
GDP	−1.008^**^ (0.360)	0.593 (0.389)	1.510 (1.133)	0.014 (0.166)
Year FE	Yes	Yes	Yes	Yes
Individual FE	Yes	Yes	Yes	Yes
Province-by-year FE	Yes	Yes	Yes	Yes
Pseudo *R*^2^	0.391	0.473	0.506	0.328
Observations	4,972	4,972	4,972	4,962

Standard errors are clustered at the city level.

^***^, ^**^, and ^*^ denote significance at the level of 1, 5, and 10%, respectively.

The CHARLS data has no direct information on current care recipients. Considering that the insured elderly with a severe disability are more likely to receive care services, we use the interaction term *LTC* × *ADL* denoted in the Methodology section to investigate the impacts of care provided by the LTCI program, and the results are shown in Table 6. As expected, the LTCI significantly improves the memory and cognition function of older people with a need for ADLs care. Care services provided by China's LTCI, including clinical care, health management guidance and rehabilitation training, offer benefits to older adults' objective health. It is also can be observed in Table 6 that LTCI has larger effects on alleviating depression of older people with difficulties in ADLs, relative to those with no LTC need. In addition, the elderly with no need for care experiences a significant increase in happiness. The reasons are as follows. The LTCI has a “peace of mind” effect on the insured people, because it alleviates their fears of expected financial and care costs (18). Another possible explanation is that the insured people with no difficulties in ADLs tend to care for their disabled family numbers and may benefit by freeing from care burdens.

**Table 6:** The effects on the mental health, by difficulties in ADLs.

**Variables**	**Memory**	**Cognition**	**CES-D**	**Happiness**
LTC	−1.141^**^ (0.530)	−0.241 (0.224)	−1.425^***^ (0.472)	0.114 (0.070)
LTC × ADL	2.780^**^ (1.228)	0.830^*^ (0.447)	−7.106^***^ (1.828)	0.361 (0.827)
ADL	−0.180 (0.140)	−0.276^***^ (0.078)	1.279^***^ (0.195)	−0.080^***^ (0.030)
Controls	Yes	Yes	Yes	Yes
Year FE	Yes	Yes	Yes	Yes
Individual FE	Yes	Yes	Yes	Yes
Province-by-year FE	Yes	Yes	Yes	Yes
Pseudo*R*^2^	0.391	0.614	0.477	0.047
Observations	4,962	4,962	4,962	4,962

Standard errors are clustered at the city level.

^***^, ^**^, and ^*^ denote significance at the level of 1, 5, and 10%, respectively.

### 5.2. Robustness test

**Figure 2:**
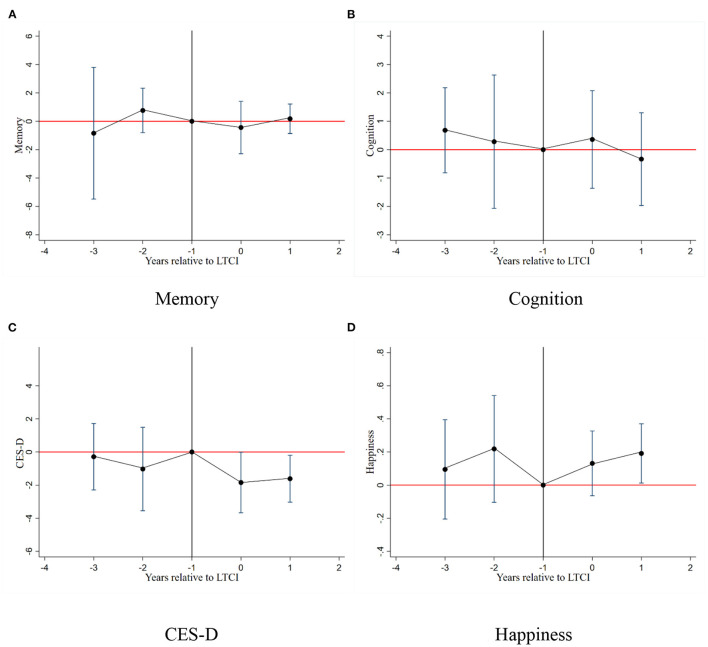
Event study for the impact on the mental health. **(A)** Memory. **(B)** Cognition. **(C)** CES-D. **(D)** Happiness.

In this section, we test the robustness of our results by five methods. First, we use the Event study (53) to test the parallel trends assumption on older adults' mental health between the treated and control groups before the implementation of China's LTCI. Figure 2 depicts the results of each year's coefficients. The reference is the year before the program (denoted by −1). It is observed that estimated results are close to 0 in the pre-reform period, implying that there are no confounding pre-existing trends in their mental health. Additionally, the CES-D and happiness experience significant changes in the post-reform period, consistent with our results in Table 4.

Second, we use the Goodman-Bacon decomposition method to examine the bias caused by time-varying treatment effects over time. According to Goodman-Bacon (54), the result of the DID method is calculated by a weighted average of all two-group estimators when the treatment occurs at different times. The incorrect use of already-treated groups as control groups may lead to biases. As shown in Table 7, the weight of the bad control group (T-Later vs. C-Earlier) is only 0.2%. The estimates of LTCI's effects on mental health mainly come from the third comparison (99%), implying our results are robust.

**Table 7:** The Goodman-Bacon decomposition.

**Treated vs. control**	**Memory**	**Cognition**	**CES-D**	**Happiness**
T-earlier vs. C-later	0.7%	0.7%	0.7%	0.8%
T-later vs. C-earlier	0.2%	0.2%	0.2%	0.2%
T vs. never treated	99.1%	99.1%	99.1%	99.0%

“T-earlier vs. C-later” means insured people in Qingdao act as the treated group and insured people in other pilot cities act as control groups. “T-later vs. C-earlier” means insured people in other pilot cities act as treated groups and insured people in Qingdao act as the control group. “T vs. never treated” means the comparison of all insured people vs. never-insured people.

Third, cities with a higher degree of aging and better social insurance systems are more likely to be selected as pilot cities, thus initial pilots may not be randomly selected. If there is no implementation of the LTCI program, the mental health outcomes of the elderly in pilot cities may be different from that of other cities, thus resulting in biased results. Therefore, we choose the cities located same provinces with pilot cities announced in 2016 and pilot cities added in 2020 as the control group. The reason is that older people living in these cities have more similar characteristics to the treated groups. The similar estimated results in Table 8 suggest that our findings are robust.

**Table 8:** The effects of LTCI on older people's mental health.

**Variables**	**Memory**		**Cognition**		**CES-D**		**Happiness**	
LTC	0.835^*^ (0.436)	−1.141^*^ (0.563)	−0.216 (0.193)	−0.287 (0.241)	−1.740^**^ (0.742)	−1.530^**^ (0.556)	0.105^***^ (0.009)	0.109 (0.067)
LTC × ADL		2.708^*^ (1.394)		1.015^**^ (0.426)		−7.280^***^ (2.096)		0.387 (0.875)
ADL		−0.277 (0.226)		−0.051 (0.117)		0.935^*^ (0.434)		−0.051 (0.045)
Controls	Yes	Yes	Yes	Yes	Yes	Yes	Yes	Yes
Year FE	Yes	Yes	Yes	Yes	Yes	Yes	Yes	Yes
Individual FE	Yes	Yes	Yes	Yes	Yes	Yes	Yes	Yes
Province-by-year FE	Yes	Yes	Yes	Yes	Yes	Yes	Yes	Yes
Observations	2,028	2,026	2,028	2,026	2,028	2,026	2,024	2,022
Pseudo*R*^2^	0.346	0.346	0.531	0.530	0.524	0.528	0.326	0.328

^***^, ^**^, and ^*^ denote significance at the level of 1, 5, and 10%, respectively.

**Figure 3:**
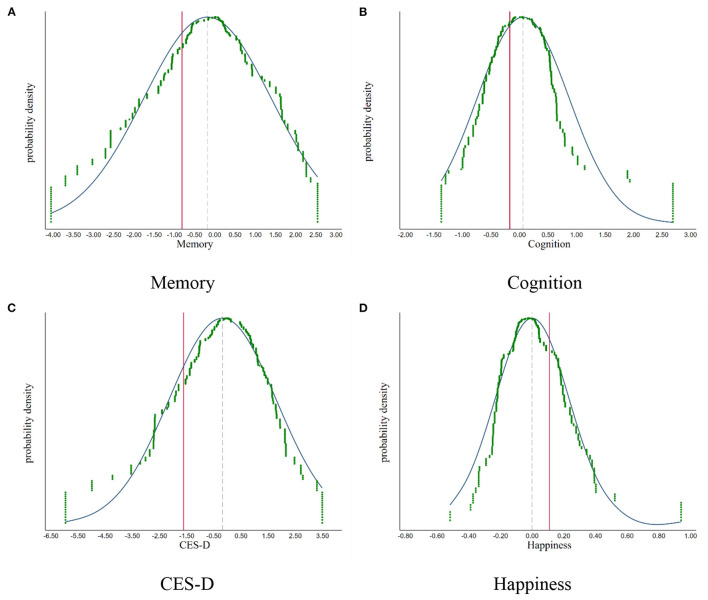
The placebo test: simulation results of the **LTC**. **(A)** Memory. **(B)** Cognition. **(C)** CES-D. **(D)** Happiness.

**Figure 4:**
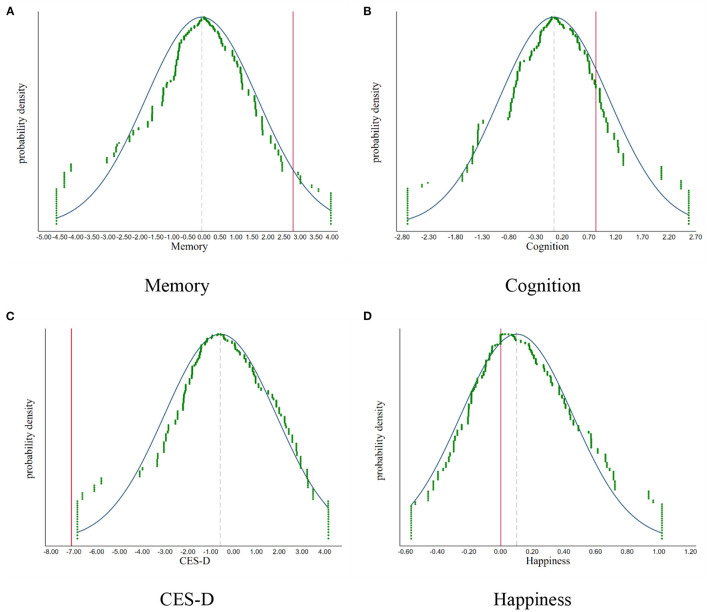
The placebo test: simulation results of the *LTC* × *ADL*. **(A)** Memory. **(B)** Cognition. **(C)** CES-D. **(D)** Happiness.

Fourth, we make a placebo test by randomly selecting a city without LTCI during 2011–2018 as the pilot city where older adults act as the treated groups. We repeat the selection 500 times and graph the density distribution of the coefficient of *LTC* and *LTC* × *ADL*. As shown in Figures 3, 4, the results of the simulation are concentrated around 0, indicating there are no other factors affecting our results. The vertical solid line and the imaginary line represent empirical results and the mean of simulation results, respectively. We find that both lines do not overlap. China's LTCI reduces depression and improves the insured people's happiness, and has positive effects on the memory and cognition function of the elderly with ADLs needs for care. Therefore, our findings are confirmed.

Fifth, we use instrumental method to solve the problem of endogeneity. The first instrumental variable selected is the number of family members living together, which are associated with older people's choice to care service types. The supply of community care service is the second instrument, which can affect the availability of LTCI benefits, while not directly related to older people's health. As shown in Table 9, both instrumental variables are valid. We can observe that LTCI reduces older people's depression symptoms and improves their life satisfaction, which confirms our findings.

**Table 9:** The effects of LTCI: IV method.

**Variables**	**Memory**	**Cognition**	**CES-D**	**Happiness**
LTC	−30.074 (16.411)	7.361 (6.587)	−37.787^**^ (18.931)	5.001^**^ (2.255)
Weak identification test: C-D F statistic	3.432^*^	2.969^*^	4.983^**^	6.130^***^
Over identification test	0.304	2.053	1.122	1.153
Sargan-Hansen test	(0.582)	(0.152)	(0.289)	(0.283)

^***^, ^**^, and ^*^ denote significance at the level of 1, 5, and 10%, respectively. The value of F statistic in the first-stage model reject the null hypothesis which indicates weak instrument variables. The results of the Sargan-Hansen test (p-values in parentheses) receive the null hypothesis that the instruments are valid.

### 5.3. Mediating effects

We find that LTCI significantly improves older people's subjective mental health. We further examine three potential channels through which LTCI benefits older people: care burdens (*Care*), the incidence of diseases (*Disease*) and community care services (*Checkup* and *Entertain*). As shown in Table 10, LTCI not only significantly reduces the care services to older adults' children and the number of diseases (columns 1 and 4), but also promotes them to receive community services (columns 7 and 10). As we all know, care burdens, diseases and community services play significant role on older people's mental health. For instance, community care services provided by LTCI in China provide health checks, rehabilitation exercises and companionship (5), which improve older people's life satisfaction. Therefore, the impacts of LTCI are partially mediated through providing community services, reducing care burdens and the incidence of diseases.

**Table 10:** The results of mediating effects.

**Variables**	**Care (1)**	**CES-D (2)**	**Happiness (3)**	**Variables**	**Disease (4)**	**CES-D (5)**	**Happiness (6)**
LTC	−0.040^*^ (0.018)	−0.770 (0.920)	0.148^**^ (0.069)	LTC	−0.102^***^ (0.034)	−1.600^**^ (0.219)	0.108^***^ (0.015)
Care		1.100^**^ (0.449)	−0.054 (0.061)	Disease		0.386^*^ (0.219)	−0.029 (0.023)
Observations	3,439	3,439	3,435	Observations	4,969	4,969	4,959
Year FE	Yes	Yes	Yes	Year FE	Yes	Yes	Yes
Individual FE	Yes	Yes	Yes	Individual FE	Yes	Yes	Yes
Province-by-year FE	Yes	Yes	Yes	Province-by-year FE	Yes	Yes	Yes
Controls	Yes	Yes	Yes	Controls	Yes	Yes	Yes
*R* ^2^	0.255	0.499	0.338	***R* ^2^**	0.764	0.517	0.336
**Variables**	**Checkups (7)**	**CES-D (8)**	**Happiness (9)**	**Variables**	**Entertain (10)**	**CES-D (11)**	**Happiness (12)**
LTC	0.835^*^ (0.436)	−0.643 (0.254)	0.073 (0.094)	LTC	0.081^**^ (0.036)	−0.026 (0.847)	0.073 (0.094)
Checkup		−0.643^**^ (0.254)	0.107^***^ (0.033)	Entertain		−0.999^*^ (0.601)	0.107^***^ (0.033)
Controls	Yes	Yes	Yes	Controls	Yes	Yes	Yes
Observations	3,154	3,055	3,103	Observations	3,154	3,055	3,103
*R* ^2^	0.022	0.187	0.069	*R* ^2^	0.016	0.186	0.069

^***^, ^**^, and ^*^ denote significance at the level of 1, 5, and 10%, respectively.

### 5.4. Heterogeneity

**Figure 5:**
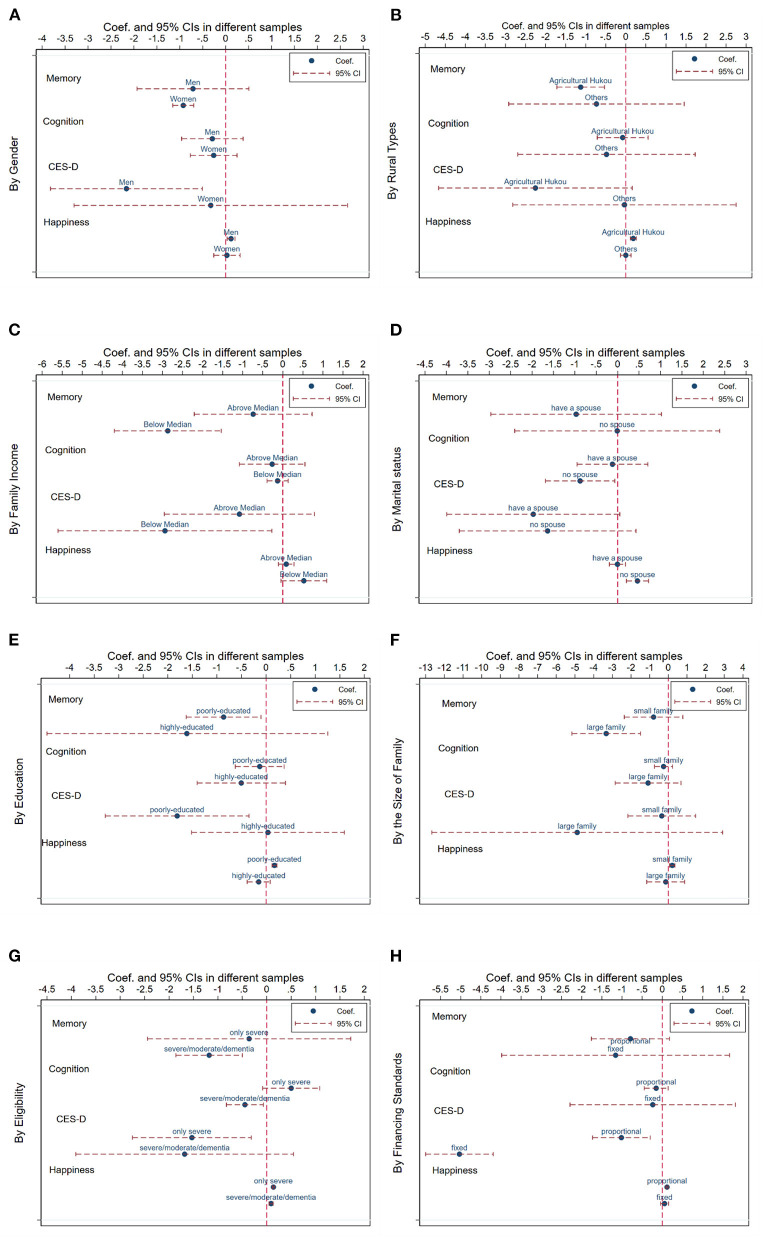
Heterogeneous impacts of LTCI. **(A)** Gender. **(B)** Rural types. **(C)** Family income. **(D)** Marital status. **(E)** Education. **(F)** Family size. **(G)** Eligibility. **(H)** Financing standard.

In this section, we compare the effects of LTCI by older adults' socioeconomic and demographic characteristics and the LTCI designs. Figure 5 depicts the respective results of the point estimates and their 95% confidence intervals, which displays unequal effects of China's LTCI. As shown in panels (A) and (B), LTCI coverage has more positive effects on their subjective mental health of men and older adults with no spouse. LTCI has higher marginal benefits to the elderly without a spouse, the loneliness of whom is higher than that of older adults who have a spouse. For the rural population, although LTCI has negative effects on their memory function, it improves their life satisfaction. Panel (D) shows that LTCI benefits both the higher-income and lower-income groups, but the positive effects are larger for lower-income adults. Lower-income and rural people are more likely to be unable to assess private formal care due to their lower affordability (55), and the results imply that China's LTCI reduces inequalities in resources between older adults in urban and rural areas or different income groups. Panel (E) and (F) reflect larger positive effects on life satisfaction of poor-educated people and people with a smaller family.

In addition, we study the difference according to the LTCI designs including its eligibility and financing standards, as shown in panels (G) and (H). The results show that the LTCI system conditional on severe disability significantly improves the cognition function of the elderly. It also confirms the moral hazard, implying that more stringent eligibility makes the elderly increasingly health-conscious, eventually reducing their cognition decline. As shown in panel (H), both financing standards are beneficial for the insured people. A fixed amount of financing more significantly reduces older people's depression symptoms.

### 5.5. Spillover effects of LTCI

Some studies have confirmed that LTCI benefits the insured people's family members, especially their caregivers (26, 56). The study on China's LTCI is still not enough. We further study the spillover effects on older adults' spouse. They are main caregivers according to the CHARLS data, implying that they are relatively healthy and are less likely to directly enjoy care services provided by LTCI. Therefore, they tend to receive spillover benefits. As shown in Table 11, although LTCI has insignificant effects on the life satisfaction of the insured people's spouses, LTCI significantly improves their physical health and stimulates their social participation. It indicates that LTCI has a positive effect on spouse caregivers' health and social isolation. The reason may be that China's LTCI improves spouse caregivers' time flexibility and allows them seek more leisure activities.

**Table 11:** The spillover effects of LTCI.

**Variables**	**Shealth**	**Shappiness**	**Social**
LTC	0.407^*^ (0.22149)	−0.004 (0.157)	0.098^***^ (0.044)
Controls	Yes	Yes	Yes
Year FE	Yes	Yes	Yes
Individual FE	Yes	Yes	Yes
Observations	3,547	3,361	3,686
*R^2^*	0.450	0.275	0.309

^***^ and ^*^ denote significance at the level of 1, 5, and 10%, respectively.

## 6. Conclusion

China has introduced LTCI pilots against the care demands of the growing aging population. This study investigates the effects of China's LTCI program on the mental health of the covered adults, those with needs for ADLs care, and the wellbeing of their families. Based on the CHARLS data in 2011, 2013, 2015, and 2018, we adopt the staggered DID approach to study the initial LTCI pilots from 2015 to 2017. We adopt a rich set of mental health indexes including subjective and objective health status from self-reported measures and biomarkers in CHARLS. We study whether and how China's LTCI affect older people's mental health, and further examine the spillover effects on their spousal caregivers covering their self-reported health, life satisfaction and social activities. Additionally, we compare these effects across different dimensions of older people's characteristics and the LTCI designs.

We find some interesting results. First, LTCI has a significant correlation with insured older adults' depression symptoms and life satisfaction. The care services provided by LTCI also improve the memory and cognition function of insured adults with ADLs needs for care, who is most likely to enjoy LTCI's services. Second, we explore the influence mechanism of older adults' mental health by providing community services, reducing care burdens and the incidence of diseases. Third, LTCI is not only beneficial to the covered people but also their caregivers. LTCI coverage improves caregivers' physical health and entertainment. Additionally, the effects of LTCI on older people's, mental health vary by the LTCI designs and older adults' socioeconomic and demographic characteristics differentials.

This study has some policy implications. First, much attention should be paid to addressing mental health problems and expanding the scope of evaluation criteria, such as mental or psychological state. Second, the ongoing LTCI has positive effects on older adults, especially vulnerable groups such as the lower-income people and rural population. However, China's LTCI currently is not very comprehensive, and only covers a narrow group. Given the important role of government spending, policymakers should expand the coverage and add more LTCI pilots. Third, it is important to promote home care and enrich the types of community services due to its role as the mediator. This is also consistent with China's elderly preference to live with their families in their own homes. Fourth, huge unmet needs still exist. For instance, China's LTCI has limited effects on women. The LTCI system should attach importance to incorporating the different needs and preferences. Additionally, policymakers could apply the big data technology to manage information about the insured people's health status and their needs to achieve efficient resource allocation. Policymakers should also focus on the response of older people to LTCI coverage, which is beneficial to reducing moral hazard, eventually improving older adults' health and happiness.

Due to data limitation, our estimated results are “intention to treat” effects measuring impacts on targeted population, which may underestimate the effects of LTCI. Moreover, this study is limited by the absence of information on what kind of care services qualified older adults received. Therefore, we could not examine differential effects of home-visit, community, and nursing/medical institutional care. We encourage future study to extend knowledge of the impacts of LTCI by collecting on more detailed data on LTC needs and service types.

## Data availability statement

Publicly available datasets were analyzed in this study. This data can be found here: http://charls.pku.edu.cn/.

## Author contributions

YC: software, data curation, writing—original draft, and visualization. HZ: conceptualization, methodology, writing—review and editing, and supervision. All authors contributed to the article and approved the submitted version.

## Footnotes

^1^The seventh national census of China.

^2^ https://population.un.org/wpp/Download/Standard/Population/
